# Rediscovering the carotid pulse: unlocking hidden insights in the era of AI-driven healthcare

**DOI:** 10.3389/fneur.2025.1608651

**Published:** 2025-06-16

**Authors:** Hilla Ben-Pazi, Shady Jhashan, Haill Salame, Hillit Cohen, Idit Matot, Meron Ben Pazi, Yaniv Kirma, Lior Elmaliach, Yair Lavi, Pnina Mintz, Nicola Corvaja, Danny Dvir, Haim Danenberg, Marc Ribo

**Affiliations:** ^1^Avertto Medical LTD, Aderet, Israel; ^2^Department of Neurosurgery, Galilee Medical Center, Nahariya, Israel; ^3^Department of Cardiac Surgery, Sheba Medical Center, Ramat Gan, Israel; ^4^Department of Anesthesiology and Critical Care and Pain, Tel Aviv Medical Center, Tel Aviv, Israel; ^5^Interventional Cardiology, New York-Presbyterian Hospital, New York, NY, United States; ^6^Columbia University Irving Medical Center, New York, NY, United States; ^7^Department of Cardiology, Shaare Zedek Medical Center, Jerusalem, Israel; ^8^Department of Cardiology, E. Wolfson Medical Center, Holon, Israel; ^9^Stroke Unit, Vall d'Hebron University Hospital, Barcelona, Spain

**Keywords:** carotid artery, artificial intelligence, monitoring, stroke, pulse wave velocity, ultrasound, baro-sensors

## Abstract

From ancient Chinese medicine to medieval European practice, the carotid pulse has long been recognized as a vital window into vascular health. Yet in modern clinical medicine, this rich physiological signal has been largely overlooked. While artificial intelligence (AI) has transformed healthcare through advanced data interpretation, it has also inadvertently diverted focus from acquiring novel physiological data—particularly from the carotid artery. This review highlights the underutilized potential of carotid hemodynamics and explores how emerging sensor technologies, combined with AI, can transform stroke prevention, real-time cerebrovascular monitoring, and broader vascular care. As a central conduit between the heart and brain, the carotid artery conveys dynamic hemodynamic information relevant not only to neurology, but also to cardiology and pulmonary medicine. Recent advances in non-invasive, continuous monitoring now enable real-time assessment of vascular stiffness, pulse wave patterns, and early cerebrovascular compromise—capabilities that were previously inaccessible with traditional, intermittent evaluation methods. Focusing on the neurological context, this review outlines emerging opportunities in carotid monitoring, identifies key hemodynamic markers, and evaluates the clinical consequences of their underuse. By integrating AI with enhanced, continuous data acquisition from the carotid artery, the medical community may pursue new diagnostic and predictive pathways, advancing toward proactive, precision-based care and improved patient outcomes.

## 1 Introduction

For centuries, the carotid pulse has served as a powerful diagnostic tool, valued across cultures—from ancient Chinese medicine to medieval European practice—for its ability to reflect internal health. Today, this long-standing physiological insight can be reimagined through modern technology: emerging tools now enable continuous, non-invasive monitoring of carotid signals. While clinical practice has historically prioritized established metrics like ECG, blood pressure, and oxygen saturation, there is now a clear opportunity to expand physiological monitoring by tapping into the underused potential of the carotid artery.

The stroke field, like many domains of medicine, is increasingly integrating artificial intelligence (AI) at multiple levels. Its most widespread application is in the interpretation of diagnostic imaging (1). Furthermore, established stroke risk factors are now embedded within AI-driven models, improving predictive accuracy beyond what traditional approaches offer (2). AI is also being applied to vascular imaging, particularly to enhance analysis of carotid artery narrowing and plaque morphology—both widely used indicators of cerebrovascular risk (3).

However, these imaging-based assessments are intermittent, offering only isolated snapshots of vascular health. This reliance on static evaluations reinforces the view of stroke as a sudden event, when it is a dynamic, escalating process—more akin to a ticking time bomb that evolves silently before symptoms emerge. Single-point assessments lack the responsiveness needed for early intervention, and even the most advanced risk prediction models are of limited value without real-time alerts.

Shifting to continuous physiological monitoring enables early detection of hemodynamic changes, reframing stroke as a process that can be detected and potentially interrupted before irreversible damage occurs. A paradigm shift is underway—from periodic risk estimation to a real-time diagnostic monitoring that can alert clinicians to pathological changes as they emerge and escalate (Table 1). This mirrors advances in cardiology, where real-time alert systems are already used to detect deterioration and guide timely care (4). A similar approach is now emerging in stroke prevention, enabled by wearable and implantable sensors paired with AI-driven signal analysis of the carotid arteries (5, 6). This review explores the untapped value of carotid hemodynamics, introduces emerging monitoring approaches, and discusses their potential to transform stroke prevention and vascular health.

**Table 1:** Moving from predictive to diagnostic markers.

Category	Examples	Clinical utility
Predictive	Arterial stiffness, vascular reactivity	Useful for risk stratification
Diagnostic	Plaque morphology, stenosis severity,	Detect pre-existing abnormalities
Real-time Monitoring	Continuous detection of hemodynamic changes	Enables early alerts for stroke prevention

## 2 Aren’t there any available monitoring tools for real time stroke detection?

While cardiac parameters are continuously monitored in clinical settings, real-time stroke monitoring remains notably limited. Despite advancements in noninvasive stroke diagnostic modalities, existing technologies have yet to consistently demonstrate early stroke detection or significant improvement in patient outcomes.

### 2.1 Traditional cardiovascular tools

Such as blood pressure monitoring, ECG, and echocardiography, provide limited real-time insights into cerebrovascular dynamics. Large-scale, AI-driven initiatives aiming to predict stroke via wearable devices, including studies by Apple and Johns Hopkins University, have not yet demonstrated clinical efficacy. To date, no results have been published from the ongoing NIH-funded trial evaluating Apple Watch-guided anticoagulation management for stroke prevention. While shifting from risk estimation toward real-time stroke detection holds theoretical promise for early intervention, clinical validation remains lacking (7). However, recent advances in AI and deep learning offer promising avenues for real-time stroke detection. For example, recent work has introduced real-time stroke detection via real-time facial paralysis detection with enhanced accuracy and privacy on edge devices (8). Other research has demonstrated deep learning-based prediction systems using real-time EEG signals, achieving high accuracy with low false positive rates (9). These emerging efforts demonstrates a growing momentum in leveraging AI for continuous stroke monitoring, complementing physiological sensors and promising to bridge existing gaps in real-time detection.

### 2.2 The bispectral index

The bispectral index (BIS) derived from EEG signals, is widely used intraoperatively to detect cerebral ischemia, but its sensitivity is limited, particularly for subcortical events. Originally developed to assess anesthesia depth, BIS shows some correlation with ischemia during carotid clamping but lacks a clinically reliable threshold for stroke detection. Consequently, BIS frequently yields false positives, limiting its standalone effectiveness (10).

### 2.3 Near-infrared spectroscopy

Provides noninvasive monitoring of cerebral oxygenation with a high negative predictive value for hypoperfusion. However, its utility is restricted by sensor placement primarily over the frontal lobes, inter-device variability, and susceptibility to extracerebral interference. Despite promising sensitivity and specificity, the absence of a universally accepted threshold limits its clinical reliability (11). A recent systematic review reported no significant advantage of near-infrared spectroscopy (NIRS) over standard monitoring regarding mortality, neuroprotection, or adverse events (12).

### 2.4 Transcranial Doppler ultrasound

Effectively detects cerebral microemboli and assesses blood flow dynamics (13). Yet, its use in continuous bedside monitoring is hindered by operator dependency, anatomical variability among patients, and the requirement for manual probe stabilization. Additionally, continuous robotic transcranial Doppler (TCD) remains limited by high costs, patient positioning challenges, the need for specialized training, and insufficient large-scale clinical validation specific to stroke detection (14).

### 2.5 Multimodal monitoring

Strategies combining different technologies might provide enhanced diagnostic accuracy and clinical utility. For instance, integrating EEG and TCD biomarkers significantly improved the prediction of delayed cerebral ischemia (DCI) in subarachnoid hemorrhage (SAH) patients (15). Further development and validation of such multimodal approaches may overcome the limitations of current single-modality tools and strengthen clinical decision-making.

## 3 Why use the carotid for brain monitoring?

Most research on carotid artery disease and stroke has focused on anatomical markers—such as luminal narrowing and plaque morphology—as the basis for clinical decision-making, especially for interventions like carotid endarterectomy and stenting (16). These structural features are readily assessed through imaging. However, there is increasing recognition that anatomical data alone may not fully capture stroke risk. Real-time hemodynamic indicators, although more difficult to acquire, could provide functionally richer insights.

The carotid artery plays a central role in maintaining cerebrovascular health. It contains baroreceptors in the carotid sinus that continuously regulate blood pressure, heart rate, and vascular tone, dynamically modulating cerebral perfusion in response to physiological (17). These mechanosensitive receptors are located at the carotid bifurcation—a region prone to turbulent flow, plaque buildup, and oscillatory shear stress—which may explain their strategic placement (18, 19).

A key but underutilized functional marker is cerebrovascular reactivity (CVR), particularly CO₂ reactivity (CO₂R). This is typically measured via changes in cerebral blood flow velocity during controlled hypercapnia (e.g., breath-holding or CO₂ inhalation). A meta-analysis by Reinhard et al. (20), involving 754 patients with carotid stenosis or occlusion, found that impaired CO₂R (pCi < 20%) was strongly associated with increased ipsilateral stroke risk. Each 10% decrease in CO₂R corresponded to a 1.64-fold increase in hazard. Importantly, this relationship held true even in asymptomatic individuals, supporting its predictive value beyond symptoms.

Although functional tests like CO₂R offer valuable insights, they are rarely used and typically limited to single-point measurements—failing to reflect the dynamic, evolving nature of stroke. Despite its potential, the functional role of the carotid artery remains underutilized. Moving from static imaging to continuous, real-time monitoring could transform the carotid into an active sentinel, enabling earlier detection and intervention before neurological damage occurs.

## 4 What are the existing sensors to use for hemodynamic carotid monitoring?

Emerging technologies are transforming carotid hemodynamic monitoring by moving beyond static imaging toward real-time, continuous assessment using a range of sensor modalities. Flow sensors such as Doppler ultrasound and magnetic flow meters measure carotid blood velocity, while pressure sensors—including piezoelectric and capacitive types—detect arterial wall displacement. Motion-based sensors, such as accelerometers and strain gauges, capture tissue vibration and deformation. Each sensor type targets a distinct physiological parameter, offering a more comprehensive understanding of cerebrovascular status (Table 2).

**Table 2:** Sensor modalities for carotid monitoring.

Modality	Measurement type	Active/passive	Cost	Advantages	Challenges
PPG	Flow	Active	$	Simple	Accuracy affected by skin color
Ultrasound (US)	Flow	Active	$$	Provides extensive detailed data	Requires precise sensor positioning
Magnetic Sensor (Hall Effect)	Flow	Passive	$$	Contactless, measurement, durable	Sensitive to external magnetic fields
Accelerometer	Motion/vibration	Passive	$	Highly sensitivity	Susceptible to motion artifacts
Strain gauge sensor (PESG)	Pressure/displacement	Passive	$$	Measures direct contact force	Requires frequent calibration
Piezoelectric	Pressure/displacement	Active	$	High sensitivity, accurate waveform	Expensive, sensitive to motion
Optical reflection sensor	Pressure/displacement	Active	$$	Non-contact measurement capability	Affected by ambient light conditions
Capacitive sensor	Pressure/displacement	Passive	$$	High sensitivity, stable signal	Requires controlled contact pressure

### 4.1 Flow-based sensors

Include ultrasound-based technologies like Doppler systems and flexible skin-adhering patches, which allow for non-invasive, real-time measurements of blood flow velocity and waveform morphology (5). A recent innovation includes the point-of-care ultrasound (POCUS) approach, which has shown promise as a rapid bedside solution. In the POCUS-LVO study, trained non-specialists successfully performed carotid ultrasound exams in under 3 min, supporting its feasibility for fast stroke triage in emergency settings (21). In addition, magnetic Hall-effect-based sensors have been explored for local Pulse wave velocity (PWV) measurement. A dual magnetic plethysmograph (MPG) probe, equipped with Hall-effect sensors and permanent magnets, was validated *in vivo* for measuring carotid artery PWV (22).

### 4.2 Pressure-based sensors

Have also shown promising results. A comparative study by Wang et al. evaluated four sensor types—piezoresistive strain gauge (PESG), piezoelectric tonometer, accelerometer, and optical reflection sensors—across multiple arterial sites, including the carotid. They found that medium contact pressure produced the most stable signals, and that tonometer and accelerometer sensors demonstrated the highest stability and reproducibility (ICC > 0.80). In contrast, optical sensors were vulnerable to ambient light interference, while PESG sensors were sensitive to variations in contact pressure.

## 5 What hemodynamic factors can we use?

Various hemodynamic parameters can be utilized for stroke risk assessment and early detection, particularly through non-invasive waveform analysis.

### 5.1 Waveform analysis

Provides several valuable metrics. Carotid flow volume has been shown to inversely correlate with atherosclerosis severity (r = −0.696) (23). Additionally, differences in end-diastolic velocity between carotid arteries measured using point-of-care ultrasound (POCUS) accurately predict large vessel occlusion (LVO), achieving high diagnostic accuracy (AUC = 0.90; specificity = 83%). Combining waveform parameters further enhances diagnostic capabilities. Shimada et al. (24) utilized differences in amplitude, timing, and shape of carotid pulse waves, applying logistic regression to detect carotid artery occlusion, achieving a diagnostic accuracy of 0.65.

### 5.2 Pulse wave velocity

The speed at which arterial pressure waves propagate—is among the most validated hemodynamic indicators. Pulse wave velocity (PWV) is an established surrogate measure of arterial stiffness, with higher values indicating reduced arterial compliance. It can be assessed regionally (e.g., carotid-femoral) or locally at the carotid artery, where local PWV provides targeted insight into arterial elasticity (23). Although PWV is not validated as a standalone diagnostic tool for stroke, it remains a strong predictive marker. Elevated PWV and carotid stiffness have consistently been associated with increased stroke risk, poor prognosis, and cognitive decline (24, 25). Notably, PWV asymmetry between the left and right carotid arteries during acute stroke episodes has uncovered diagnostic differences that were not detected by standard flow velocity alone (26).

Collectively, waveform shape, bilateral asymmetry, flow volume, carotid stiffness, and PWV form a robust set of hemodynamic biomarkers, providing essential tools for non-invasive cerebrovascular assessment and enabling AI-driven stroke prediction and prevention strategies (27, 28).

## 6 What are the main challenges?

The integration of artificial intelligence (AI) and advanced sensor technologies for continuous carotid monitoring offers substantial promise for proactive stroke prevention, but several significant challenges must be overcome to realize this potential fully.

### 6.1 Technical developmental challenges

Developing reliable and precise carotid monitoring systems requires overcoming considerable technical complexities. High-resolution sensors capable of continuous, accurate measurements of carotid hemodynamics are challenging to design and produce, demanding substantial engineering resources and iterative research and development (R&D) processes. Transitioning these technologies from laboratory prototypes to clinically usable devices involve extensive testing, validation, and refinement, often placing significant financial and logistical burdens on research institutions and commercial enterprises.

Furthermore, maintaining device performance and ensuring user comfort during prolonged monitoring periods adds complexity. Wearable sensors must balance accuracy, usability, durability, and patient compliance, necessitating multidisciplinary collaboration across engineering, medical, and design teams.

### 6.2 Data acquisition

While retrospective datasets currently dominate AI applications in healthcare—particularly diagnostic imaging—they inherently lack the dynamic, real-time physiological data critical for conditions like acute stroke. Capturing these real-time carotid waveforms and integrating them into predictive models poses substantial hurdles. Continuous data acquisition demands dedicated equipment and ongoing infrastructure investments. Additionally, existing databases, often commercially valuable and readily accessible, reduce incentives to generate new physiological datasets, resulting in fewer resources allocated toward novel data collection.

Moreover, even when collected, new physiological signals frequently remain disconnected from existing electronic medical records (EMRs), limiting their availability for clinical decision-making and large-scale analytics. Overcoming this barrier requires deliberate and coordinated efforts to integrate new physiological data streams into healthcare systems and clinical workflows.

### 6.3 Regulatory and ethical challenges

Current regulatory frameworks often lag rapid advancements in AI and sensor-based technologies. Novel diagnostic tools utilizing AI-driven continuous monitoring systems must navigate complex and often outdated regulatory pathways. These frameworks were not initially designed to handle the pace of innovation inherent to AI technologies, causing significant delays, uncertainty, and increased costs during the approval process.

Ethical considerations also pose significant challenges. Issues regarding patient privacy, data security, informed consent, and the ethical use of AI-generated insights must be thoroughly addressed. Transparent and robust ethical frameworks are needed to manage patient data responsibly, ensuring trust and acceptance among healthcare professionals and patients.

### 6.4 Clinical integration and adoption challenges

Integrating continuous carotid monitoring systems into clinical practice presents numerous practical hurdles. Healthcare providers must adopt established workflows to incorporate new technologies, which can disrupt routines and create resistance among medical staff. Additionally, uncertainties regarding the clinical utility, reliability, and overall value of these novel systems may slow adoption.

To effectively promote clinical adoption, extensive validation studies involving diverse patient populations are necessary. These studies must clearly demonstrate not only the clinical efficacy and safety of continuous carotid monitoring but also its cost-effectiveness and practicality in real-world healthcare settings. Ongoing education and training for healthcare providers will be crucial in facilitating smooth transitions and ensuring optimal use of these technologies.

### 6.5 Economic challenges

Developing, validating, and implementing novel sensor-based AI technologies entail substantial financial investments. High upfront costs associated with research, device manufacturing, and regulation can deter funding and slow innovation, especially when compared to more immediate returns from existing data-driven solutions. Additionally, reimbursement strategies for diagnostic innovations often lag, further complicating the economic viability of these novel tools.

Securing sustainable financial support from both private and public sectors, along with favorable reimbursement policies, is essential to enable continued development, validation, and widespread adoption of continuous carotid monitoring technologies.

## 7 Conclusion

Advancements in AI and sensor technologies present unprecedented opportunities for transforming stroke prevention strategies from reactive management to proactive, real-time intervention. Historically valued for its enhanced risk stratification, and precision-based preventive care the carotid pulse now offers even greater potential through modern technological innovations. Continuous carotid monitoring holds considerable promise in providing critical physiological insights, enabling earlier stroke detection. Moving toward systematic expansion of real-time physiological monitoring can significantly improve stroke outcomes. However, achieving this requires addressing and overcoming the significant technical, regulatory, ethical, clinical, and economic challenges outlined above. Comprehensive collaboration among researchers, healthcare providers, regulatory agencies, and policy-makers is essential to overcome these barriers and unlock the full potential of continuous carotid monitoring, ultimately improving patient outcomes and substantially reducing stroke-related morbidity and mortality.
